# Functional and structural insights into the multicopper oxidase MmcO from *Mycobacterium tuberculosis*: implications for drug targeting

**DOI:** 10.3389/fchem.2025.1565715

**Published:** 2025-05-27

**Authors:** Dafeng Liu, Feng Yu, Yihan Luo, Ayitunihe Hanate

**Affiliations:** ^1^ Xinjiang Key Laboratory of Lavender Conservation and Utilization, College of Biological Sciences and Technology, Yili Normal University, Yining, Xinjiang, China; ^2^ School of Life Sciences, Xiamen University, Xiamen, Fujian, China

**Keywords:** tuberculosis (TB), *Mycobacterium tuberculosis* (MTB), multicopper oxidase MmcO, site-specific mutation, enzymatic activity assays

## Abstract

*Mycobacterium tuberculosis* (*Mtb*) is a significant and highly pathogenic intracellular microorganism responsible for tuberculosis (TB). The global TB crisis has been exacerbated by the emergence and spread of multidrug-resistant *Mtb* strains, resulting in elevated mortality rates. *Mtb* MmcO exhibits scavenging activity against reactive oxygen species (ROS), thereby supporting *Mtb* survival. However, the molecular mechanism underlying MmcO function remains poorly understood. Herein, the hydrodynamic radius of MmcO was determined to be 5.9 ± 0.3 nm. A structural model of MmcO was predicted using AlphaFold2 and subsequently evaluated for accuracy using a Ramachandran plot and ProSA analysis. Site-directed mutagenesis revealed that substitutions H120A, H122A, H161A, or H163A nearly abolished the activity, while mutations H120R, H122R, H161R, or H163R led to minor alterations in the activity. The addition of Triton X-100 or Ca^2+^ significantly enhanced MmcO activity, whereas EDTA or other metal ions markedly inhibited its activity to varying extents. MmcO, a multicopper oxidase, plays a role in maintaining redox homeostasis in *Mtb*, a function critical for bacterial survival in host macrophages. Our study reveals that Cu^2+^ is essential for enzymatic activity, while Ni^2+^, Mn^2+^, and Zn^2+^ inhibit function, likely due to improper metal coordination. Given its importance in oxidative stress resistance, MmcO presents a promising drug target for *Mtb* therapy. Therefore, this study offers valuable insights for developing novel therapeutic agents targeting *Mtb*.

## Introduction

Tuberculosis (TB) is a persistent infectious disease caused by *Mycobacterium tuberculosis* (*Mtb*) and remains a significant global health challenge (Getahun et al., 2015; Reid et al., 2019; Pai et al., 2016; Dowdy and Behr, 2022; Furin et al., 2019). According to the World Health Organization, despite the extensive implementation of intensive chemotherapy over the past several decades, approximately one-quarter of the global population remains infected with *Mtb* (https://www.who.int/news-room/fact-sheets/detail/tuberculosis). TB severity is exacerbated by co-morbid conditions, such as infections with HIV or SARS-CoV-2 (Dheda et al., 2022; Flores-Lovon et al., 2022; Udoakang et al., 2023; Dhana et al., 2022; Rule et al., 2021). The emergence of drug-resistant *Mtb* strains poses a major challenge to TB treatment, which exhibit resistance to key anti-TB drugs, including isoniazid, pyrazinamide, and rifampicin (Dominguez et al., 2023; Naidoo and Dookie, 2022; Dean et al., 2022). These drug-resistant strains significantly compromise the efficacy of TB treatment, resulting in elevated mortality rates (Tiberi et al., 2018; Micoli et al., 2021; Lange et al., 2019; Jenkins et al., 2014; Getahun et al., 2015; Dheda et al., 2017; Furin et al., 2019; Reid et al., 2019). Therefore, a deeper understanding of *Mtb* pathogenesis is urgently required to facilitate the development of more effective therapeutic strategies against TB.

Multicopper oxidases play key roles in bacterial pathogenesis, with homologs in *Pseudomonas aeruginosa* and *Salmonella enterica* contributing to copper detoxification and oxidative stress resistance. In *Mtb*, MmcO may function similarly to SodA and KatG in mitigating reactive oxygen and nitrogen species, highlighting its potential as a novel drug target.

*Mtb* has the ability to evade host immune surveillance and clearance, allowing it to persist within host macrophages for extended periods (O’Garra et al., 2013; Getahun et al., 2015; Tiberi et al., 2018). Within macrophages, *Mtb* is exposed to diverse oxidative stress conditions, including fluctuations in pH, reactive oxygen species (ROS), enzymatic degradation, nutrient deprivation, and nitrogen intermediates (Aderem and Underhill, 1999; Agarwal et al., 2021; Kinkar et al., 2019; Sharma et al., 2023; Raya et al., 2022). *Mtb* MmcO has been shown to exhibit ROS scavenging activity, effectively neutralizing ROS produced in the xanthine-xanthine oxidase enzyme system and, more critically, ROS generated in activated THP-1 cells (Kinkar et al., 2019). The cysteine residue in the N-terminus of *Mtb* MmcO aligns with a putative signal peptidase cleavage site and likely serves as the site for lipid modification that anchors the protein to the membrane. MmcO represents a promising target for the development of novel therapeutic agents against *Mtb*. However, the functional mechanisms and detailed molecular roles of *Mtb* MmcO are not well known.

Here, the structural model of MmcO was predicted using AlphaFold2 and subsequently evaluated using a Ramachandran plot and ProSA. The results of site-directed mutagenesis experiments demonstrated that substitutions H120A, H122A, H161A, or H163A nearly abolished enzymatic activity, while mutations H120R, H122R, H161R, or H163R caused minor alterations in the activity. The addition of Triton X-100 or Ca^2+^ significantly enhanced MmcO activity, whereas EDTA or other metal ions markedly reduced activity to varying extents. These findings highlight two critical sites on MmcO essential for its activity and provide intriguing insights into the metal ion preferences of *Mtb* MmcO. These offers a potential foundation for the development of novel antitubercular therapies.

## Results

### Bioinformatics analysis

**FIGURE 1:**
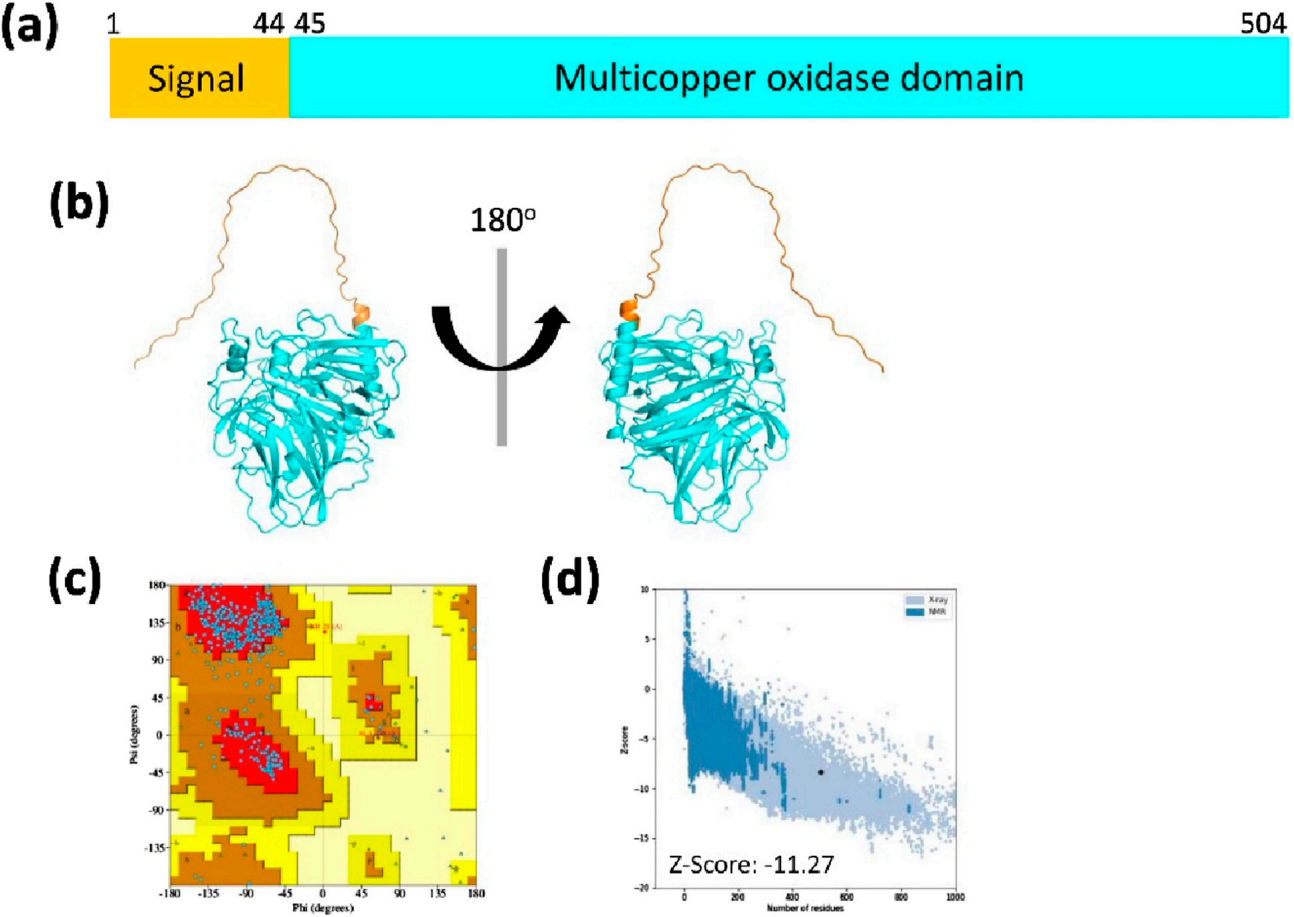
The cluster organization and structural model of MmcO. **(a)** Schematic diagram illustrating the signal (orange) and multicopper oxidase (cyan) domains. **(b)** The MmcO structure, predicted using AlphaFold2, is shown in ribbon representation from two orientations, with the conserved domain colored as depicted in Figure 1A. **(c)** Structural validation of MmcO was carried out using Ramachandran Plot analysis, where the most favored regions are highlighted in red, with progressively lighter shades indicating less favored regions. **(d)** ProSA analysis indicated that the Z-score of the MmcO structure was −11.27.

The multicopper oxidase MmcO of *Mtb* contains a single conserved domain, the multicopper oxidase domain, spanning amino acids 45–504 (Figure 1a; Supplementary Figure S1). The sequence of MmcO was obtained from the UniProt database (accession number I6WZK7). The estimated molecular weight of MmcO is approximately 53.8 kDa. Its molecular formula was determined to be C_2386_H_3717_N_667_O_715_S_19_, and its isoelectric point (pI) was calculated to be 5.9.

The efficacy of codon optimization was evaluated by analyzing the codon adaptation index (CAI) and GC content. The optimized CAI for MmcO was determined to be 84.6% (Table 1). The GC content, which is considered optimal within the range of 30%–70%, was optimized to 58.3% for MmcO, falling well within the acceptable range (Table 1).

**TABLE 1:** Codon optimization of MmcO.

Optimization for codon	Codon adaptation index (CAI) value	GC content value
Before codon optimization	67.2%	64.0%
After codon optimization	84.6%	58.3%

### Prediction and quality assessment of MmcO structure

The three-dimensional (3D) structure of MmcO was predicted using the AlphaFold2 (Jumper et al., 2021; Wayment-Steele et al., 2023; Tunyasuvunakool et al., 2021) (Figure 1b). In contrast to traditional homology modeling approaches utilized in earlier studies, AlphaFold2 leverages deep learning techniques to achieve improved accuracy and reliability in protein structure prediction (Supplementary Figure S2).

The quality of the predicted MmcO structural model was evaluated using a Ramachandran plot, which assessed whether the dihedral angles of the protein backbone were within acceptable regions, thereby validating the structural integrity of the model (Figure 1c; Supplementary Figure S2). The analysis revealed that 86.9% of the residues were located in the most favored regions, 12.6% in the additionally allowed regions, 0.5% in the generously allowed regions, and none in the disallowed regions (Figure 1c; Table 2). Furthermore, the structure included 2 terminal residues, 55 glycine residues, and 41 proline residues (Figure 1c; Table 2). The quality of structural model was further confirmed using ProSA, an interactive web tool for detecting errors in 3D protein structures, which provided a Z-score of −11.27, indicating the high quality of the predicted structure (Figure 1d).

**TABLE 2:** Ramchandran plot analysis of MmcO structural model using PDBsum.

Constructs	Residues in most favored regions		Residues in additional allowed regions		Residues in generously allowed regions		Residues in disallowed regions	
Residues	Number of residues	% of residues^*a*^	Number of residues	% of residues	Number of residues	% of residues	Number of residues	% of residues
Statistics	353	86.9	51	12.6	2	0.5	0	0

Note: Number of end-residues (excl. Gly and Pro): 2; Number of glycine residues (shown as triangles): 55; Number of proline residues: 41.

Overall, the structural model of MmcO is of good quality and is suitable for further following analysis.

### Characterization of MmcO by dynamic light scattering

**FIGURE 2:**
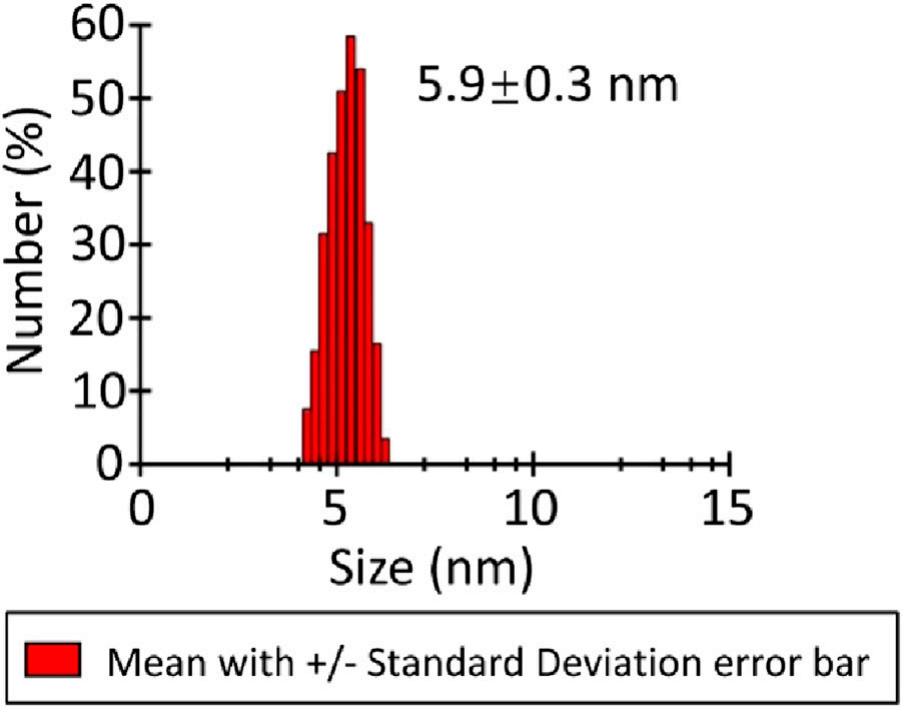
Dynamic light scattering (DLS) spectrum of MmcO. The hydrodynamic radius of MmcO was measured as 5.9 ± 0.3 nm, showing the existence of monomeric MmcO.

Dynamic light scattering (DLS) experiments were conducted to further examine the oligomeric state of MmcO by determining its diameter following centrifugation. The regularized DLS histograms were analyzed, showing that the hydrodynamic radius of MmcO was 5.9 ± 0.3 nm (Figure 2). This result indicated that MmcO existed in a monomeric form.

### MmcO crystals resisted optimization

**FIGURE 3:**
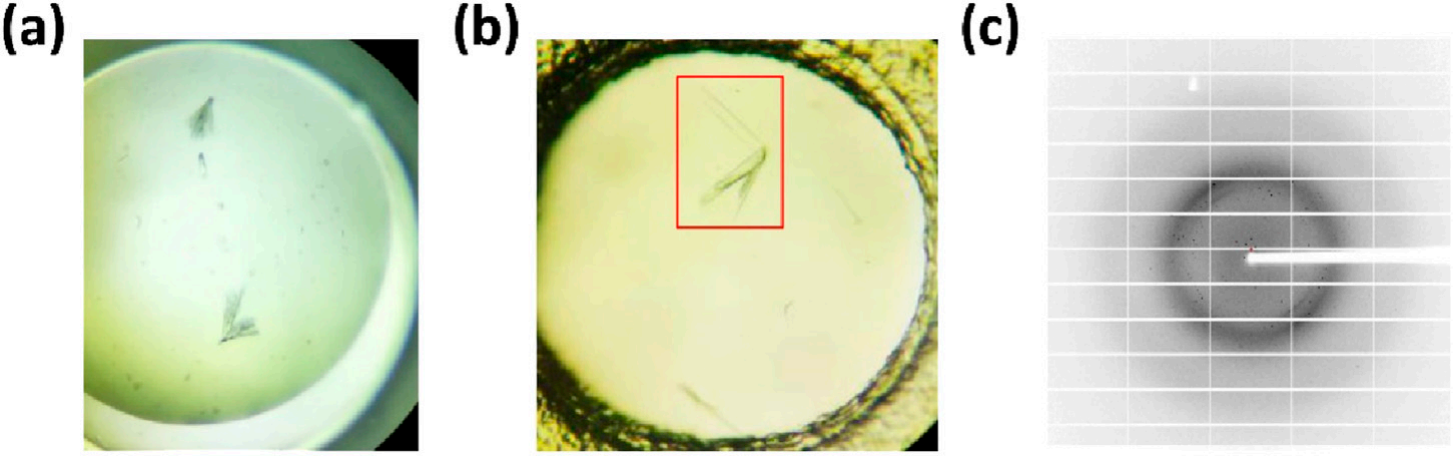
The crystal showed limitations in obtaining optimized diffracting MmcO crystals. **(a)** MmcO crystals were of poor quality. **(b)** Despite extensive optimization efforts, attempts to improve the crystallization of MmcO^83-504^ (in red frame) were unsuccessful in producing suitable diffraction patterns. Consequently, **(c)** the diffraction image obtained for MmcO^83-504^ was of low quality, with few clearly resolvable diffraction spots.

The primary objective of this study was to elucidate the structure of full-length MmcO in order to investigate the relationship between its functional and structural characteristics. However, despite considerable effort, optimization of MmcO crystallization proved unsuccessful (Figure 3a).

Following initial crystal screening of three recombinant MmcO proteins (MmcO^45-504^ and MmcO^83-504^), crystals of MmcO^83-504^ were successfully obtained in 8% (w/v) polyethylene glycol (PEG) 3,350 with 0.1 M sodium formate at pH 4.5. However, despite extensive efforts to optimize the crystallization conditions (Figure 3b), the MmcO^83-504^ crystals showed poor diffraction quality and were resistant to further optimization (Figure 3c). Consequently, crystal optimization remains ongoing.

### MmcO^83-504^ accounts for the full catalytic activity of full-length MmcO

**FIGURE 4:**
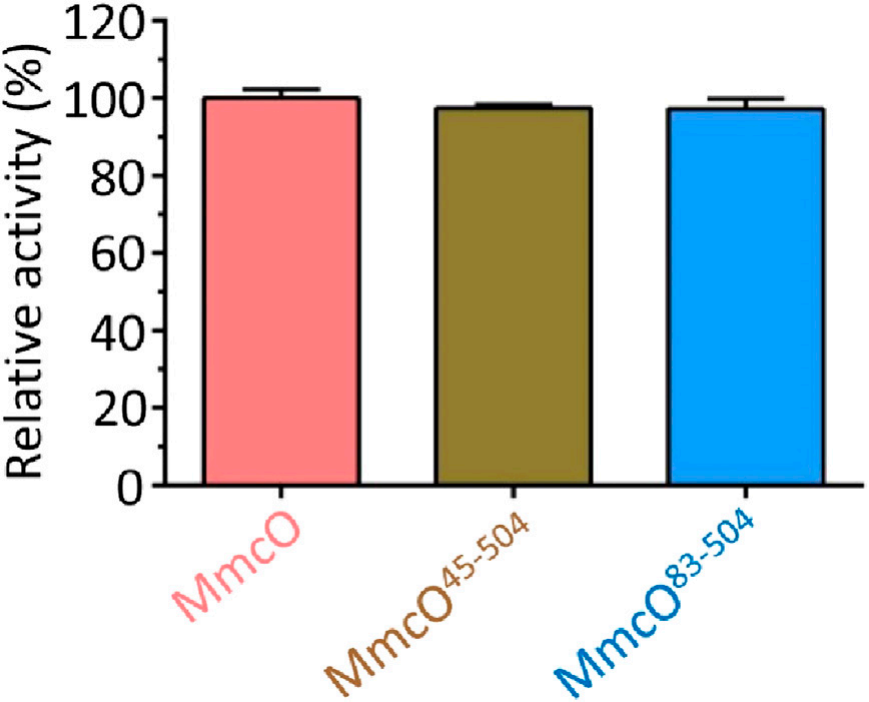
Comparative analysis of the multicopper oxidase activity between MmcO^45-504^, MmcO^83-504^ and MmcO. The multicopper oxidase activity of MmcO^45-504^ or MmcO^83-504^ was identical to that of full-length MmcO, showing that MmcO^45-504^ or MmcO^83-504^ was responsible for all full-length MmcO activity. The activity of wild-type (WT) MmcO was set to 100%.

Full-length MmcO was truncated based on the structural model predicted using AlphaFold2 (Tunyasuvunakool et al., 2021; Jumper et al., 2021; Tunyasuvunakool et al., 2021; Wayment-Steele et al., 2023; Abramson et al., 2024). However, the specific role of MmcO^45-504^ or MmcO^83-504^ in regulating the multicopper oxidase activity of the full-length MmcO remained unclear. Both MmcO^45-504^ and MmcO^83-504^ were expressed and purified using the same protocols as full-length MmcO. Our results showed that the copper oxidase activity of MmcO^45-504^ and MmcO^83-504^ was identical to that of the full-length MmcO (Figure 4), demonstrating that MmcO^83-504^ plays a crucial role in maintaining the activity of the full-length MmcO.

### Enzymatic activity for MmcO site-directed mutation

**FIGURE 5:**
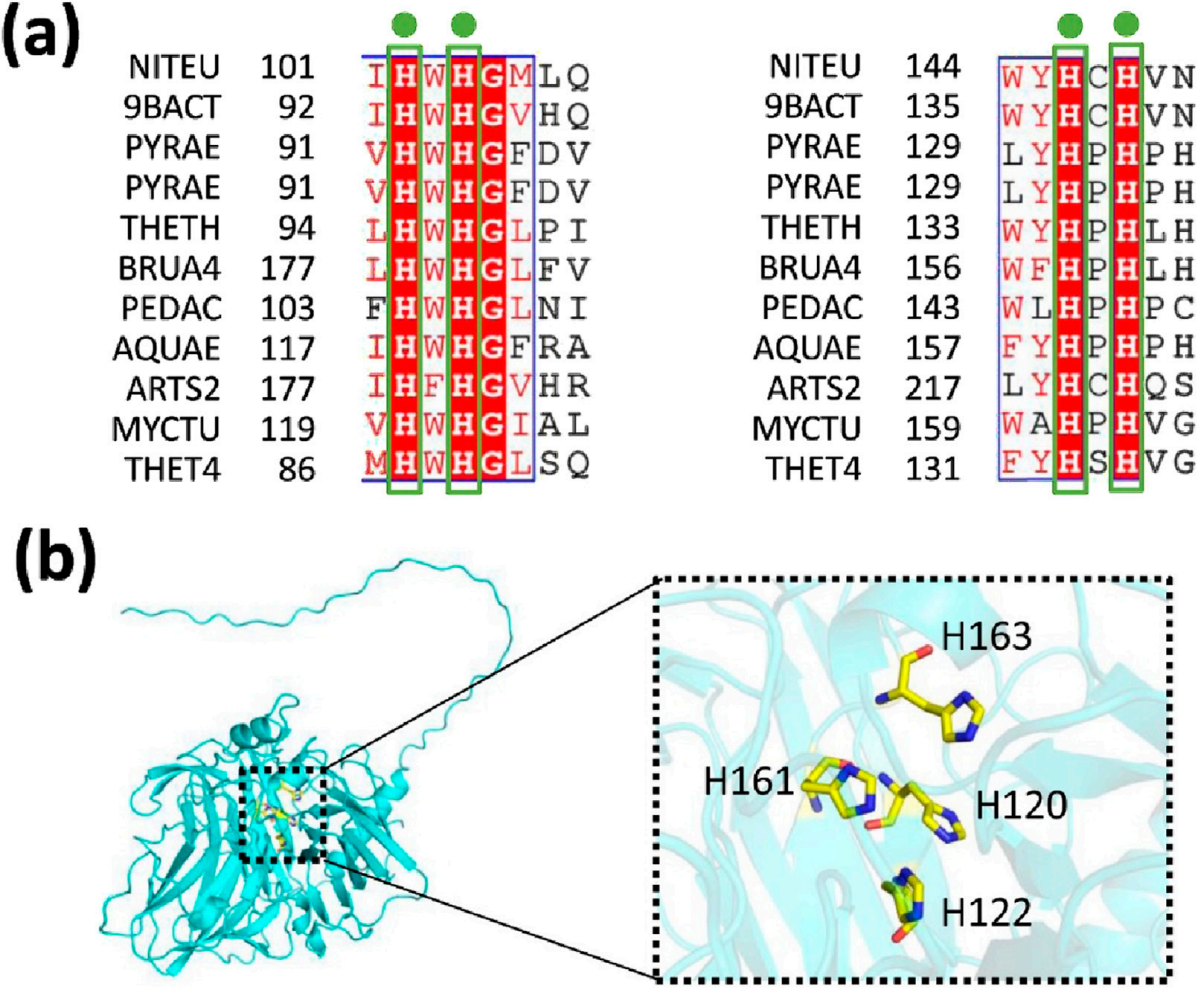
Relative multicopper oxidase activities of wild-type (WT) MmcO and its indicated mutants. **(a)** Sequence alignment of conserved residues from various species. NITEU, *Nitrosomonas europaea*; 9BACT, *Escherichia coli*; PYRAE, *hyperthermophilic archaeon Pyrobaculum aerophilum*; PYRAE, *Pyrobaculum aerophilum* str. IM2; THETH, *Thermus thermophilus*; BRUA4, *Ochrobactrum*; PEDAC, *Pediococcus acidilactici*; AQUAE, *hyperthermophile aquifex aeolicus*; ARTS2, *Arthrobacter* sp. FB24; MYCTU, *Mycobacterium tuberculosis; THET4*, *Thermothelomyces thermophilus*. **(b)** The conserved amino acids are identified as: H120, H122, H161 and H163. These conserved residues are highlighted with sticks and colored yellow. The structural model of MmcO is displayed in a cyan cartoon format.

**FIGURE 6:**
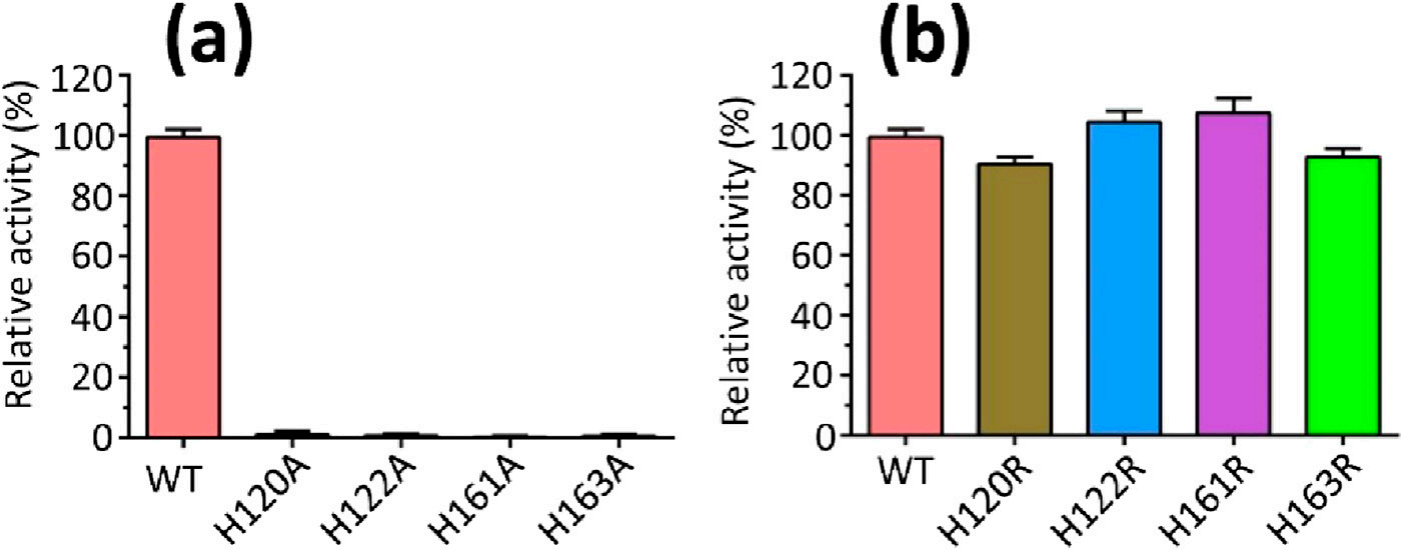
Relative multicopper oxidase activities of wild-type (WT) MmcO and its indicated mutants. **(a)** H120A, H122A, H161A or H163A almost completely abolished the activity, whereas, **(b)** H120R, H122R, H161R or H163R caused slight change in the activity compared to WT protein. The OD (optical density) value at 412 nm was measured using ABTS (2,2′-azino-bis-3-ethylbenzthiazoline-6-sulphonic acid). The activity of wild-type (WT) MmcO was set to 100%.

Mutagenesis experiments were performed based on the sequence alignment results (Figure 5; Supplementary Figure S1). The mutations H120A, H122A, H161A, or H163A resulted in the complete loss of copper oxidase activity when compared to the wild-type (WT) protein (Figure 6a). In contrast, mutations H120R, H122R, H161R, or H163R caused only minor changes in activity (Figure 6b). This result can be attributed to the stabilizing effects of His and Arg residues, which possess positive charges. Consequently, residues H120, H122, H161, and H163 are crucial for MmcO activity, and their proximity to the substrate underscores the importance of these mutations.

### Effects of metal ions, EDTA and TritonX-100 on MmcO activity

**FIGURE 7:**
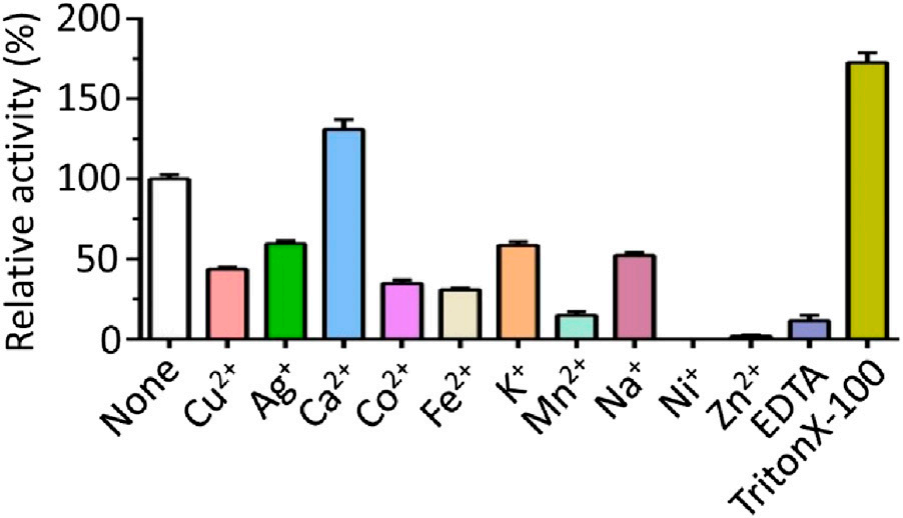
Effects of TritonX-100, EDTA and metal ions on MmcO activity. Triton X-100 or Ca^2+^ dramatically increased the activity of MmcO, whereas EDTA or other metal ions significantly decreased the activity to varying degrees. The OD value was measured at 412 nm. The activity of WT (wild-type) MmcO without addition of metal ions or chelators was set to 100%.

The influence of metal ions on MmcO activity was investigated (Figure 7). MmcO activity was significantly inhibited by Ni^+^ (Nickel), Mn^2+^ (Manganese), and Zn^2+^ (Zinc), with inhibition exceeding 80% (Figure 7). Co^2+^ (Cobalt), Cu^2+^ (Copper), Fe^2+^ (Ferrous), K^+^ (Potassium), Na^+^ (Sodium), and Ag^+^ (Silver) ions also inhibited MmcO activity to varying degrees (40%–70% inhibition) (Figure 7). In contrast to these inhibitory effects, Ca^2+^ (Calcium) ions enhanced MmcO activity by 130% (Figure 7). The inhibition of MmcO by Ni^2+^, Mn^2+^, and Zn^2+^ is likely due to their competitive displacement of Cu^2+^ at the active site, thereby impairing electron transfer efficiency. Furthermore, pre-treatment with EDTA (Ethylene diamine tetraacetic acid) before dialysis resulted in a 90% reduction in MmcO activity (Figure 7), implying the essential role of metal ions in MmcO function. Treatment with Triton X-100 led to a 172% increase in activity (Figure 7), suggesting that MmcO may be membrane-associated.

## Discussion

In this study, we determined the hydrodynamic radius of MmcO to be 5.9 ± 0.3 nm. The structural model of MmcO was predicted using AlphaFold2 and subsequently evaluated with the Ramachandran plot and ProSA. Based on sequence alignment results, we performed mutagenesis experiments and found that mutations H120A, H122A, H161A, or H163A almost completely abolished MmcO activity, while mutations H120R, H122R, H161R, or H163R caused only minor alterations in activity. Treatment with Triton X-100 or Ca^2+^ significantly enhanced MmcO activity, whereas EDTA and other metal ions inhibited activity to varying extents. Our findings identified key residues involved in catalytic activity, providing valuable insights for the development of new therapeutic agents targeting *Mycobacterium tuberculosis* (*Mtb*).

Due to the failure to obtain MmcO crystals, we conducted a more comprehensive investigation into its functional mechanism. To assist in this analysis, we employed SWISS-MODEL (https://Swissmodel.expasy.org/) to identify structural homologs of MmcO (Supplementary Table S1). Our search revealed that MmcO exhibited amino acid sequence identities of 36.13%, 33.41%, 33.16%, 32.95%, and 32.91% with terpene synthases from uncultured bacterium, *Canariomyces arenarius*, *Aspergillus niger*, *Thermus thermophilus*, and *Pseudomonas thermotolerans*, respectively (Supplementary Table S1). These findings offer valuable insights into the structural and functional mechanisms of MmcO in *Mtb*.

The preservation of residual activity in histidine-to-arginine mutants suggests that MmcO’s active site has compensatory electron transfer pathways. Further structural studies, including molecular dynamics simulations, are needed to explore these adaptive mechanisms.

Given its role in oxidative stress defense, MmcO is a promising therapeutic target. Inhibitors designed to disrupt Cu^2+^ binding, similar to existing copper-chelating antibiotics, could enhance *Mtb* susceptibility to host immune responses. To conclude, our study offers a new perspective for exploring the complex functional mechanisms of MmcO in *Mtb*.

## Materials and methods

### Bioinformatics analysis

The predicted amino acid sequence of MmcO (UniProt accession number I6WZK7) was analyzed using the ProtParam online server (https://web.expasy.org/protparam/) to assess its chemical properties and physicochemical parameters. All gene sequences were optimized according to a previously described method (Liu et al., 2025b) using the online tool (https://www.novopro.cn/tools/codon-optimization.html).

### Protein construction, expression and purification

MmcO constructs, expression and purification were as described previously (Kinkar et al., 2019).

### Structure prediction and quality assessment of MmcO

The three-dimensional (3D) structure of MmcO was predicted using the AlphaFold2 program (Jumper et al., 2021; Tunyasuvunakool et al., 2021; Wayment-Steele et al., 2023). The MmcO sequences were obtained from the UniProt database under entry ID I6WZK7. Structural visualizations were created using PyMOL 2.3.4 (https://www.pymol.org/2/).

To validate the tertiary structures, PDBsum (https://www.ebi.ac.uk/thornton-srv/databases/pdbsum/Generate.html) was employed to generate the Ramachandran plot for MmcO. This tool is instrumental in the quality assessment and validation of protein structures, as it identifies geometric errors and helps ensure the accuracy of the models. Additionally, the Ramachandran plot examines the stereochemical properties of the structures by evaluating the dihedral angles of the constituent amino acid residues. The plot identifies the allowed regions for residue positioning and highlights disallowed orientations.

In parallel, ProSA (Protein Structure Analysis) is a widely used tool for protein structure validation, primarily employed to analyze and verify predicted protein models (Wiederstein and Sippl, 2007). The Z-score value reflects the overall quality of the model. Its value is presented in a plot that includes the Z-scores of all experimentally determined protein chains in the current PDB. In this plot, structures from different sources (e.g., X-ray, NMR) are differentiated by color. The Z-score can be used to determine whether the score of the input structure falls within the typical range observed for native proteins of comparable size.

### Dynamic light scattering experiments

To investigate the oligomeric state, MmcO diameter was measured using dynamic light scattering (DLS) with a Dynapro DLS instrument (Malvern Zetasizer, Malvern, United Kingdom), following previously established protocols (Liu et al., 2024a; Liu et al. 2024b; Liu et al. 2025a). The protein was concentrated to approximately 2.7 mg/mL and then subjected to centrifugation at 12,000 rpm for 5 min. MmcO was subsequently placed into a 1-cm path length cuvette, and data acquisition was carried out over 30 runs, with an equilibration period of 120 s. The resulting DLS data were analyzed using the Zetasizer software (Ver. 6.20), and regularized DLS histograms were generated. The diameter was continuously monitored throughout the analysis.

### Enzymatic activity assays of wild-type MmcO

The MmcO activity assays were performed based on the oxidation of ABTS (2,2′-azino-bis-3-ethylbenzthiazoline-6-sulphonic acid), following previously described methods with some modifications (Kinkar et al., 2019; Park et al., 1999). The assay was conducted in a 50 mM sodium acetate buffer (pH 4.0) (Sigma-Aldrich, City of Saint Louis, State of Missouri, United States) containing 2 mM ABTS (Sigma-Aldrich, City of Saint Louis, State of Missouri, United States) and 2 mM CuSO_4_ (Sigma-Aldrich, City of Saint Louis, State of Missouri, United States). The reaction mixture was incubated with shaking for 2 h. The optical density (OD) at 412 nm was measured, and the specific activity was calculated using the molar absorptivity coefficient ε = 18,400 M^-1^cm^-1^.

### Enzymatic activity assays for site-directed mutagenesis MmcO

Primers for site-directed mutagenesis of MmcO were designed in our lab and synthesized by Shanghai Sangon Biotechnology (Shanghai, China) (Tables 3, 4; Supplementary Figures S3–S4). The plasmid pET21a containing the gene encoding MmcO was served as the template, and PCR was carried out using Q5 polymerase (New England Biolabs, Ipswich, Massachusetts, United States) for the site-directed mutagenesis (Tables 3, 4; Supplementary Figures S3–S4). The success of the mutagenesis was confirmed through nucleotide sequencing by Shanghai Sangon Biotechnology (Shanghai, China). The mutated MmcO genes were then inserted into the pET21a vector. Expression and purification were conducted using the same protocols as for the wild-type (WT) MmcO. Additionally, enzymatic assays of the site-directed mutagenesis proteins were performed under conditions identical to those used for WT MmcO.

**TABLE 3:** Primers used for constructions of MmcO plasmids.

Constructs	Primers	Primer sequence (5′-3′)
pET21a-MmcO	Forward primer	TCC​GTC​GAC​AAG​CTT​ATG​CCG​GAA​CTG​GCG​ACT​TCT​GG
	Reverse primer	CTC​GAG​TGC​GGC​CGC​TCA​CAG​GAT​ATA​GTC​CAG​ACG​GGT​AGC​CAT​GC
pET21a-MmcO^45-504^	Forward primer	TCC​GTC​GAC​AAG​CTT​GCG​GGC​ATG​ACC​GCG​GC
	Reverse primer	CTC​GAG​TGC​GGC​CGC​TCA​CAG​GAT​ATA​GTC​CAG​ACG​GGT​AGC​CAT​GC
pET21a-MmcO^83-504^	Forward primer	TCC​GTC​GAC​AAG​CTT​GTG​TCT​ACC​CTG​ACC​TAC​GGT​AAT​ACC​ATT​CCG
	Reverse primer	CTC​GAG​TGC​GGC​CGC​TCA​CAG​GAT​ATA​GTC​CAG​ACG​GGT​AGC​CAT​GC

Note: The endonuclease restriction sites were underlined and displayed in bold.

**TABLE 4:** Primers used for generating site-directed mutants of MmcO.

Primers	Primer sequence (5′-3′)
H120A (F)	CTA​GCG​TA **gca** TGG​CAC​GGT​ATT​GCT​CTG​CG
H120A (R)	CGT​GCC​A **tgc** TAC​GCT​AGT​TGG​ATC​ACC​CAG
H122A (F)	GTA​CAC​TGG **gca** GGT​ATT​GCT​CTG​CGT​AAC​GAT​ATG​GAT​G
H122A (R)	GCA​ATA​CC **tgc** CCA​GTG​TAC​GCT​AGT​TGG​ATC​ACC
H161A (F)	CTG​GGC​T **gca** CCT​CAT​GTA​GGC​CTG​CAA​G
H161A (R)	CAT​GAG​G **tgc** AGC​CCA​GTA​AGT​ACC​CGG​ATC​C
H163A (F)	CTC​ACC​CT **gca** GTA​GGC​CTG​CAA​GGC​GAC
H163A (R)	CAG​GCC​TAC **tgc** AGG​GTG​AGC​CCA​GTA​AGT​ACC​C
H120R (F)	CTA​GCG​TA **cgt** TGG​CAC​GGT​ATT​GCT​CTG​CG
H120R (R)	CGT​GCC​A **acg** TAC​GCT​AGT​TGG​ATC​ACC​CAG
H122R (F)	GTA​CAC​TGG **cgt** GGT​ATT​GCT​CTG​CGT​AAC​GAT​ATG​GAT​G
H122R (R)	GCA​ATA​CC **acg** CCA​GTG​TAC​GCT​AGT​TGG​ATC​ACC
H161R (F)	CTG​GGC​T **cgt** CCT​CAT​GTA​GGC​CTG​CAA​G
H161R (R)	CAT​GAG​G **acg** AGC​CCA​GTA​AGT​ACC​CGG​ATC​C
H163R (F)	CTC​ACC​CT **cgt** GTA​GGC​CTG​CAA​GGC​GAC
H163R (R)	CAG​GCC​TAC **acg** AGG​GTG​AGC​CCA​GTA​AGT​ACC​C

Note: Mutated regions in the sequence are underlined and bold.

### Statistical analysis

Experiments were performed at least three times, and the results are presented as mean ± SD. Statistical analysis was performed using Origin 8.5, Microsoft Excel 2013, and SPSS 19.0. Statistical significance was determined by the *p*-value; *p* < 0.05 and *p* < 0.01 were considered to be significant and highly significant, respectively.

## Data Availability

The datasets generated for this study can be found in online repositories. The names of the repositories and accession numbers can be found in the article and its Supplementary Material.
