# Supplementary material for: Functional and structural insights into the multicopper oxidase MmcO from Mycobacterium tuberculosis: implications for drug targeting
Source: Front Chem. 2025 May 27;13:1565715. doi: 10.3389/fchem.2025.1565715 (PMC12149110; doi:10.3389/fchem.2025.1565715)
Supplement: Supplementary file 1 [file DataSheet2.pdf]

This website uses cookies. By continuing to browse this site, you are agreeing to the use of our site cookies. To find out more, see our Terms of Use. OK

PDBsum entry bol7

Multicopper oxidase mmco PDB id bol7

Generate full PROCHECK analyses

Contents
Protein chain
504 a.a.

PROCHECK summary for bol7

Ramachandran plot

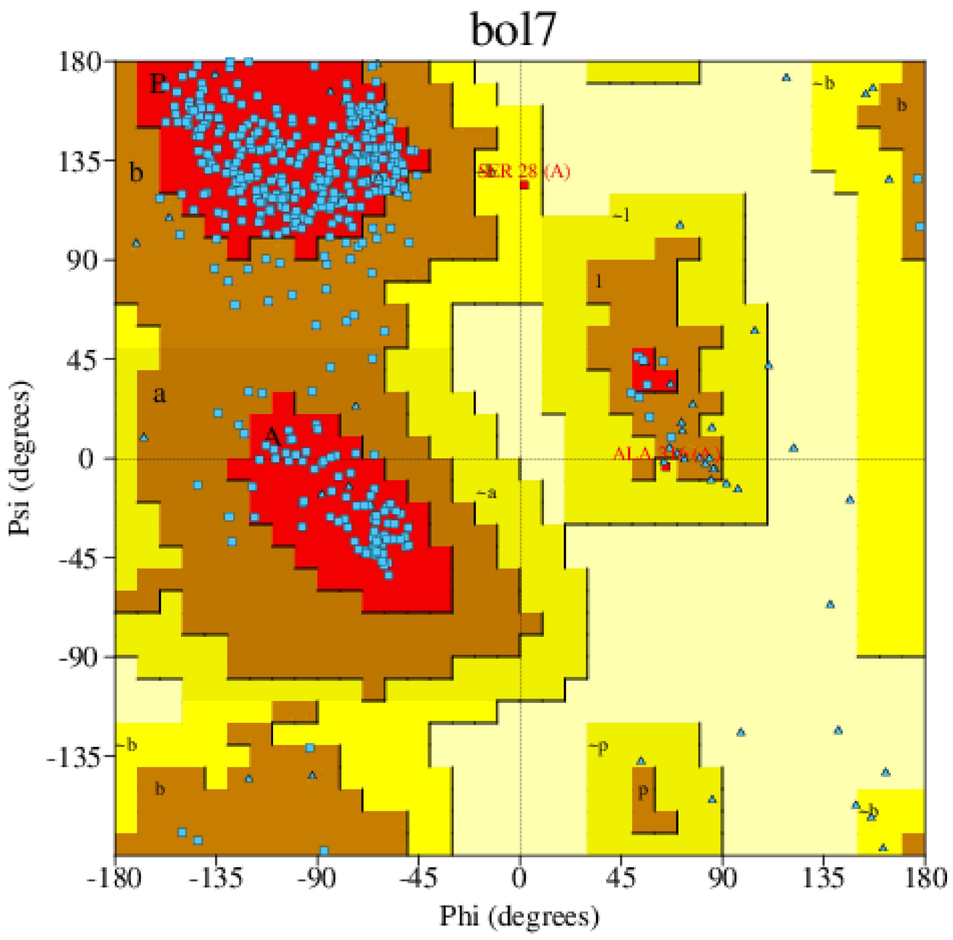

PROCHECK statistics

1. Ramachandran Plot statistics

		No. of residues	%-tage
Most favoured regions	[A,B,L]	353	86.9%*
Additional allowed regions	[a,b,l,p]	51	12.6%
Generously allowed regions	[~a,~b,~l,~p]	2	0.5%
Disallowed regions	[XX]	0	0.0%
Non-glycine and non-proline residues		406	100.0%
End-residues (excl. Gly and Pro)		2	
Glycine residues		55	
Proline residues		41	
Total number of residues		504	

Based on an analysis of **118** structures of resolution of at least **2.0** Angstroms and *R*-factor no greater than **20.0** a good quality model would be expected to have over **90%** in the most favoured regions [A,B,L].

2. G-Factors

Parameter	Score	Average Score
Dihedral angles:-		
Phi-psi distribution	-0.51*	
Chi1-chi2 distribution	0.18	
Chi1 only	0.09	
Chi3 & chi4	0.68	
Omega	-0.91*	
		-0.39
Main-chain covalent forces:-		

```
Main-chain bond lengths           0.58
Main-chain bond angles           -0.00
                                               0.24
                                              =====

OVERALL AVERAGE                               -0.12
                                              =====
```

G-factors provide a measure of how **unusual**, or out-of-the-ordinary, a property is.

```
Values below -0.5*  - unusual
Values below -1.0** - highly unusual
```

Important note: The main-chain bond-lengths and bond angles are compared with the Engh & Huber (1991) ideal values derived from small-molecule data. Therefore, structures refined using different restraints may show apparently large deviations from normality.

EMBL-EBI

News
Our impact
Contact us
Intranet

Services

By topic
By name (A-Z)
Help & Support

Research

Overview
Publications
Research groups
Postdocs & PhDs

Training

Overview
Live training
On-demand training
Support for trainers
Contact organisers

Industry

Overview
Members Area
Workshops
SME Forum
Contact Industry programme

About us

Overview
Leadership
Funding
Background
Collaboration
Jobs
People & groups
News
Events
Visit us
Contact us

EMBL-EBI, Wellcome Genome Campus, Hinxton, Cambridgeshire, CB10 1SD, UK +44 (0)1223 49 44 44

Copyright © EMBL-EBI 2024 | EBI is an outstation of the European Molecular Biology Laboratory | Terms of use
