# Supplementary material for: Functional and structural insights into the multicopper oxidase MmcO from Mycobacterium tuberculosis: implications for drug targeting
Source: Front Chem. 2025 May 27;13:1565715. doi: 10.3389/fchem.2025.1565715 (PMC12149110; doi:10.3389/fchem.2025.1565715)
Supplement: Supplementary file 2 [file DataSheet3.docx]

**Supplementary Material**

**Functional and structural insights into the multicopper oxidase MmcO from *Mycobacterium tuberculosis*: implications for drug targeting**

Dafeng Liu^1,2,*^, Feng Yu^1^, Yihan Luo^1^ and Ayitunihe Hanate^1^

^1^Xinjiang Key Laboratory of Lavender Conservation and Utilization, College of Biological Sciences and Technology, Yili Normal University, Yining 835000, Xinjiang, China;

^2^School of Life Sciences, Xiamen University, Xiamen 361102, Fujian, China.

^*^Correspondence: [dafeli@sina.com](mailto:dafeli@sina.com) or dafeli-dafeli@foxmail.com

**Table S1.**

**Table S1. Search for structural homologs of MmcO using SWISS-MODEL**

| Rank | PDB code | Identity (%) | Method | Oligo State | Ligands | Description |
| --- | --- | --- | --- | --- | --- | --- |
| 1 | 4f7k | 36.13 | X-ray, 2.2 Å | monomer | None | Laccase; Crystal structure of Lac15 from *uncultured bacterium* |
| 2 | 3pps | 33.41 | X-ray, 2.5 Å | monomer | 2xNAG-NAG, 1xNAG-NAG-BMA-MAN-MAN-MAN, 4xCU, 1xOXY, 3xNAG | Laccase; Crystal structure of an ascomycete fungal laccase from *Thielavia arenaria* |
| 3 | 5lwx | 33.16 | X-ray, 1.5 Å | monomer | 2xNAG-NAG, 4xCU, 4xNAG, 2xBMA, 1xMAN, 1xPER | Multicopper oxidase; Crystal structure of the H253D mutant of McoG from A*spergillus niger* |
| 4 | 6wcn | 32.95 | X-ray, 1.7 Å | monomer | 2xCU | Laccase; Crystal structure of Laccase from *Thermus thermophilus HB27* with an open conformation of beta-hairpin (Average deposited dose 18.08 MGy) |
| 5 | 6vow | 32.91 | X-ray, 1.9 Å | monomer | 1xC2O, 2xCU | multicopper oxidase; Crystal structure of multi-copper oxidase from *Pseudomonas Thermotolerans* |

**Figure S1.**


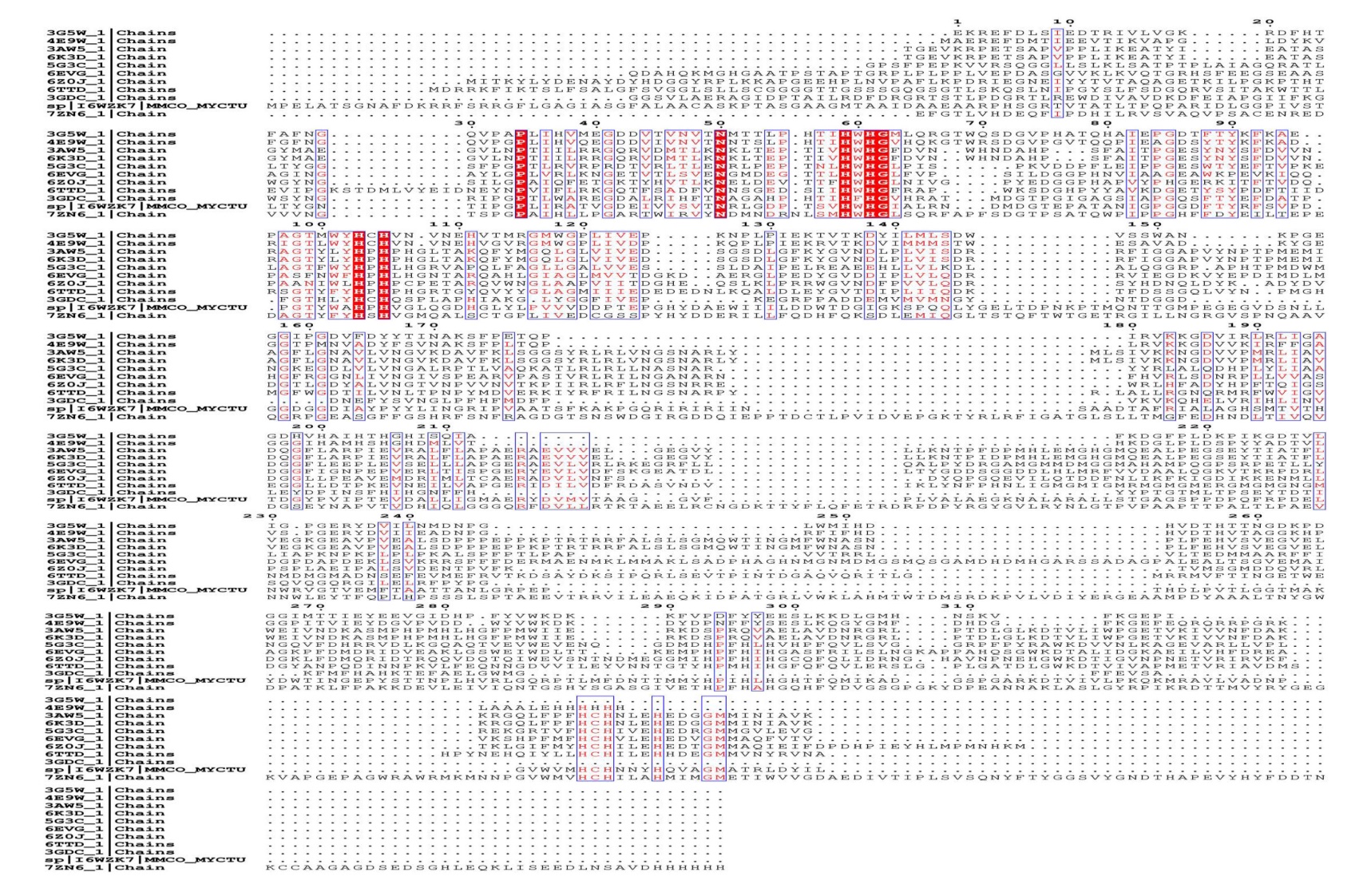


**Figure S1. Comparison of MmcO with other proteins of multicopper oxidase family.** The color scheme used is the ClustalW default scheme, with the colors for conserved amino acids being more intense than those for non-conserved ones. The following stealth proteins were included in the alignment: I6WZK7, which corresponds to MmcO from *Mycobacterium tuberculosis*; PDB code 3G5W, blue copper oxidase from *Nitrosomonas europaea*; PDB code 4E9W, multicopper oxidase mgLAC from *Escherichia coli*; PDB code 3AW5, multicopper oxidase from *hyperthermophilic archaeon Pyrobaculum aerophilum*; PDB code 6K3D, multicopper oxidase from *Pyrobaculum aerophilum* str. IM2; PDB code 5G3C, multicopper oxidase from *Thermus thermophilus*; PDB code 6EVG, multi-copper oxidase from *Ochrobactrum*; PDB code 6Z0J, laccase from *Pediococcus acidilactici*; PDB code 6TTD, multicopper oxidase from *hyperthermophile aquifex aeolicus*; PDB code 3GDC, multicopper oxidase from *Arthrobacter sp. FB24*; PDB code 7ZN6, multicopper oxidase from *Thermothelomyces thermophilus*.

**Figure S2.**


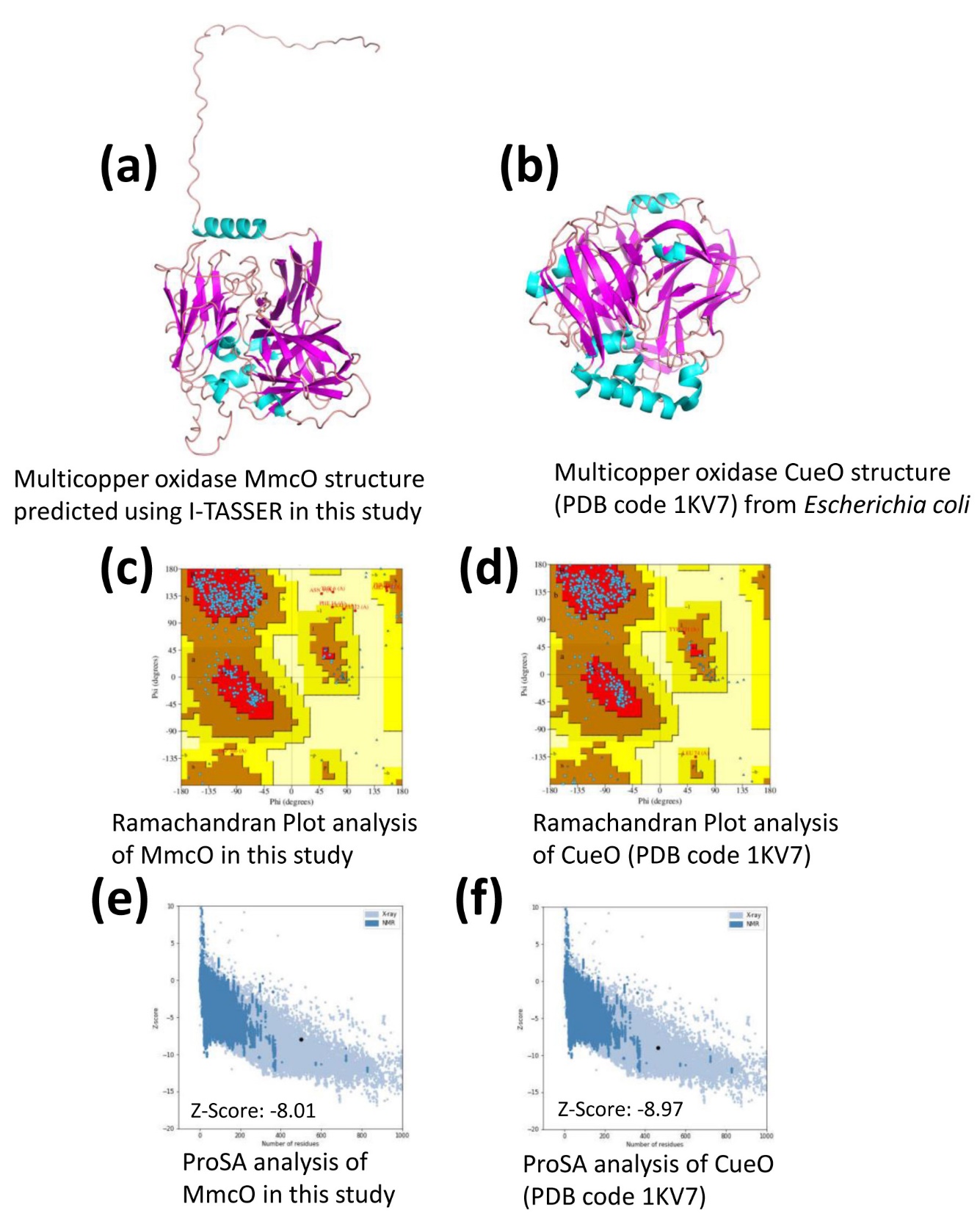


**Figure S2. Multicopper oxidase structural model prediction and quality assessment from different species.** (a) Structural model of multicopper oxidase MmcO was predicted using I-TASSER in this study. (b) Structure of the multicopper oxidase CueO (PDB code 1KV7) from *Escherichia coli*. (c) MmcO structure predicted using I-TASSER in this study and (d) CueO structure (PDB code 1KV7) were validated using Ramachandran Plot analysis, respectively, where the most favored regions are highlighted in red, with progressively lighter shades indicating less favored regions. ProSA analysis showed that Z-scores of (e) MmcO structure predicted using I-TASSER in this study and (f) CueO structure (PDB code 1KV7) were -8.01 and -8.97, respectively.

**Figure S3.**

ATGCCGGAACTGGCGACTTCTGGTAACGCTTTTGACAAACGCCGCTTCTCTCGTCGTGGTTTTCTGGGTGCCGGCATCGCTTCTGGTTTCGCACTGGCCGCGTGTGCATCTAAACCAACTGCGTCCGGCGCGGCGGGCATGACCGCGGCTATTGATGCCGCAGAGGCGGCCCGCCCGCACTCTGGTCGTACTGTGACTGCTACCCTGACCCCACAGCCGGCTCGCATCGACCTGGGTGGTCCGATTGTGTCTACCCTGACCTACGGTAATACCATTCCGGGCCCTCTGATCCGTGCAACTGTGGGCGACGAAATCGTGGTTAGCGTGACTAACCGTCTGGGTGATCCAACTAGCGTACACTGGCACGGTATTGCTCTGCGTAACGATATGGATGGCACCGAACCTGCTACTGCTAACATCGGTCCGGGTGGCGATTTCACTTACCGCTTTAGCGTTCCGGATCCGGGTACTTACTGGGCTCACCCTCATGTAGGCCTGCAAGGCGACCACGGCCTGTACCTGCCGGTAGTCGTAGACGACCCGACTGAGCCAGGTCACTACGACGCGGAGTGGATTATCATTCTGGATGACTGGACCGACGGTATTGGCAAAAGCCCACAGCAGCTGTATGGCGAGCTGACTGATCCGAACAAGCCGACTATGCAAAACACCACCGGCATGCCTGAAGGCGAAGGTGTTGATTCCAACCTGCTGGGTGGCGATGGTGGCGACATCGCGTACCCATACTACCTGATCAATGGTCGTATTCCGGTGGCGGCTACTTCTTTCAAAGCGAAACCGGGCCAGCGTATTCGTATCCGTATCATCAACTCCGCGGCGGATACCGCCTTTCGTATTGCGCTGGCAGGTCACTCCATGACCGTTACGCATACCGACGGTTACCCGGTGATCCCGACTGAAGTCGACGCGCTGCTGATCGGTATGGCGGAGCGTTACGACGTTATGGTGACGGCGGCTGGTGGTGTCTTTCCGCTGGTGGCGCTGGCTGAGGGTAAAAACGCACTGGCGCGTGCCCTGCTGTCCACTGGTGCTGGCAGCCCTCCGGACCCACAATTTCGTCCGGACGAACTGAACTGGCGCGTGGGTACTGTTGAAATGTTCACCGCTGCTACCACCGCAAACCTGGGTCGTCCGGAACCTACGCACGACCTGCCGGTGACTCTGGGCGGCACTATGGCTAAATACGATTGGACCATCAACGGTGAACCGTATAGCACGACCAATCCTCTGCATGTTCGTCTGGGTCAGCGTCCGACCCTGATGTTCGACAACACCACCATGATGTACCACCCGATCCACCTGCACGGCCACACCTTCCAGATGATCAAAGCTGACGGTTCTCCAGGTGCCCGCAAAGACACCGTGATTGTCCTGCCGAAACAGAAAATGCGTGCAGTGCTGGTCGCGGACAACCCGGGTGTATGGGTGATGCACTGCCACAACAACTACCACCAGGTTGCTGGCATGGCTACCCGTCTGGACTATATCCTG

**Figure S3. Gene sequence of *MmcO* after codon optimization**

**Figure S4.**

MPELATSGNAFDKRRFSRRGFLGAGIASGFALAACASKPTASGAAGMTAAIDAAEAARPHSGRTVTATLTPQPARIDLGGPIVSTLTYGNTIPGPLIRATVGDEIVVSVTNRLGDPTSVHWHGIALRNDMDGTEPATANIGPGGDFTYRFSVPDPGTYWAHPHVGLQGDHGLYLPVVVDDPTEPGHYDAEWIIILDDWTDGIGKSPQQLYGELTDPNKPTMQNTTGMPEGEGVDSNLLGGDGGDIAYPYYLINGRIPVAATSFKAKPGQRIRIRIINSAADTAFRIALAGHSMTVTHTDGYPVIPTEVDALLIGMAERYDVMVTAAGGVFPLVALAEGKNALARALLSTGAGSPPDPQFRPDELNWRVGTVEMFTAATTANLGRPEPTHDLPVTLGGTMAKYDWTINGEPYSTTNPLHVRLGQRPTLMFDNTTMMYHPIHLHGHTFQMIKADGSPGARKDTVIVLPKQKMRAVLVADNPGVWVMHCHNNYHQVAGMATRLDYIL

**Figure S4. Amino acid sequence of MmcO**
