# Supplementary material for: Functional and structural insights into the multicopper oxidase MmcO from Mycobacterium tuberculosis: implications for drug targeting
Source: Front Chem. 2025 May 27;13:1565715. doi: 10.3389/fchem.2025.1565715 (PMC12149110; doi:10.3389/fchem.2025.1565715)
Supplement: Supplementary file 3 [file DataSheet1.pdf]

ProSA-web
Protein Structure Analysis

Please upload a structure in PDB format: HELP

选择文件 未选择任何文件

Alternatively you can specify a structure by entering its PDB code, chain identifier and NMR model number:

PDB CODE:

PDB CHAIN ID:

PDB MODEL NUMBER:

If you leave the fields for chain id or model number blank, the first chain of the first model found in the PDB file will be analysed.

Analyse

Results for MmcO.pdb, chain A, model 1 (504 aa)

Overall model quality HELP

Z-Score: **-8.31**

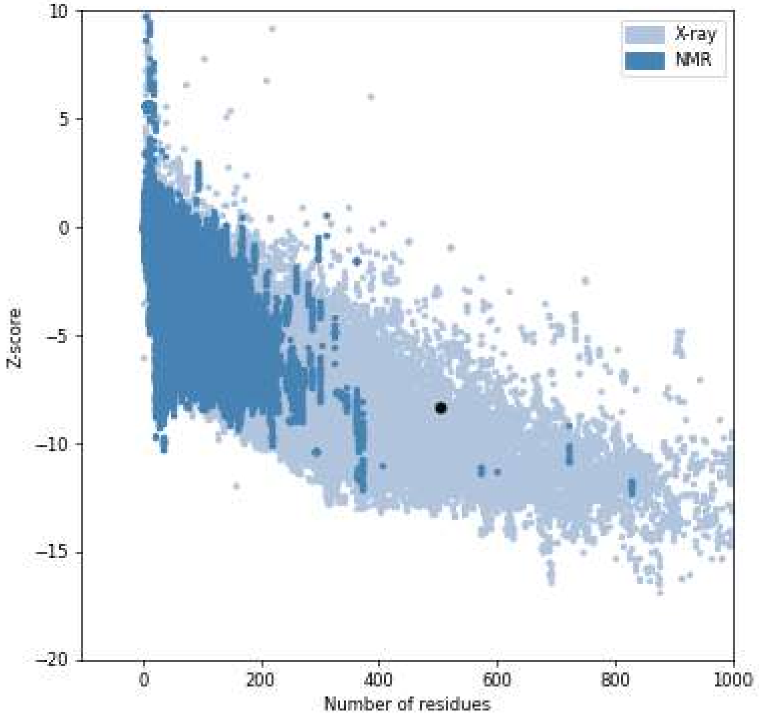

Local model quality

HELP
PNG

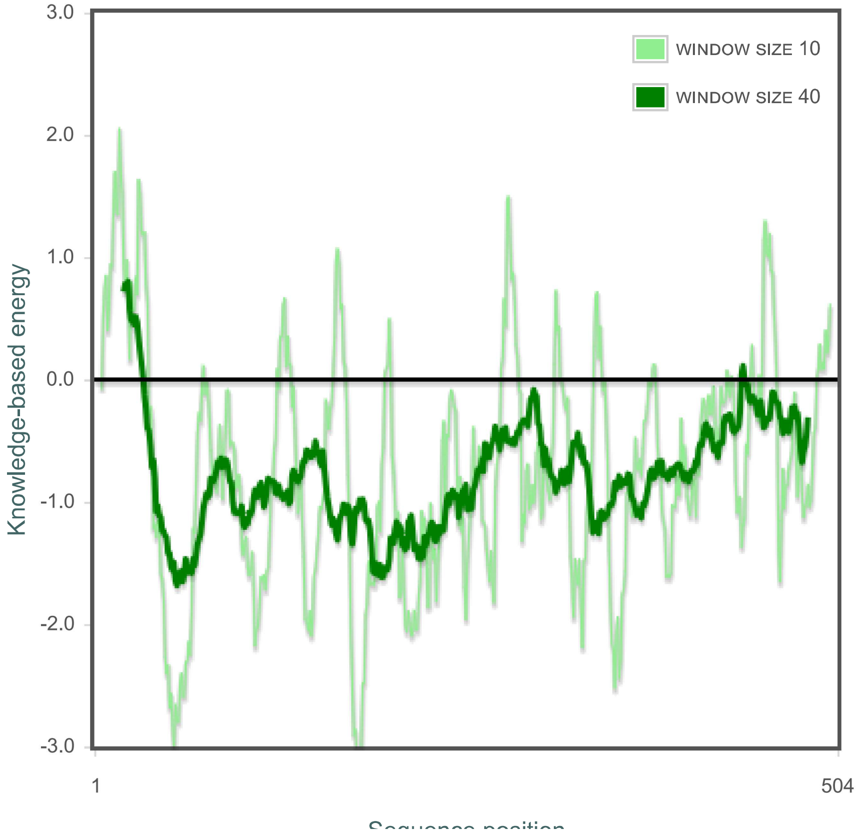

Sequence position

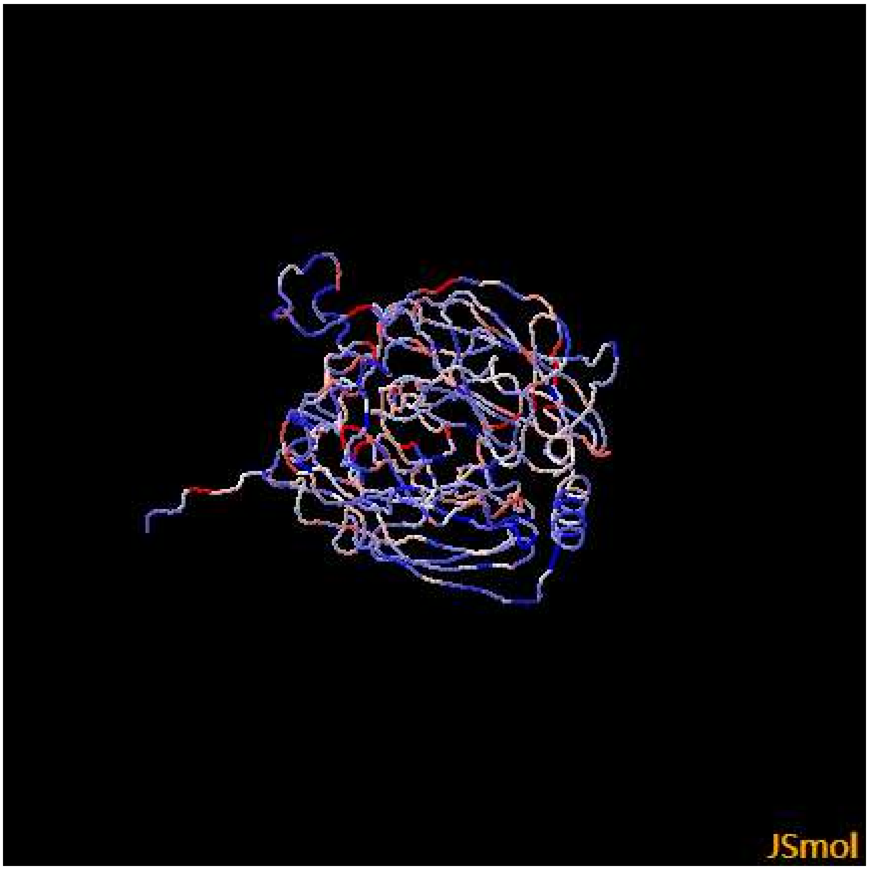

Lowest energy Highest energy

Please cite the following articles if you publish results using ProSA-web:

- Wiederstein & Sippl (2007)
  ProSA-web: interactive web service for the recognition of errors in three-dimensional structures of proteins.
  Nucleic Acids Research 35, W407-W410. [view]
- Sippl, M.J. (1993)
  Recognition of Errors in Three-Dimensional Structures of Proteins.
  Proteins 17, 355-362. [view]

This site is maintained by Markus Wiederstein. For comments and suggestions please contact prosa@came.sbg.ac.at.
